# Gene expression profiling reveals distinct features of various porcine adipose tissues

**DOI:** 10.1186/1476-511X-12-75

**Published:** 2013-05-24

**Authors:** Chaowei Zhou, Jie Zhang, Jideng Ma, Anan Jiang, Guoqing Tang, Miaomiao Mai, Li Zhu, Lin Bai, Mingzhou Li, Xuewei Li

**Affiliations:** 1 Institute of Animal Genetics & Breeding, College of Animal Science & Technology, Sichuan Agricultural University, Ya’an, Sichuan, 625000, China

**Keywords:** Gene expression profiling, Subcutaneous adipose tissues, Visceral adipose tissues, Pig

## Abstract

**Background:**

The excessive accumulation of body fat is a major risk factor to develop a variety of metabolic diseases. To investigate the systematic association between the differences in gene expression profiling and adipose deposition, we used pig as a model, and measured the gene expression profiling of six variant adipose tissues in male and females from three pig breeds which display distinct fat level.

**Results:**

We identified various differential expressed genes among breeds, tissues and between sexes, and further used a clustering method to identify sets of functionally co-expression genes linked to different obesity-related phenotypes. Our results reveal that the subcutaneous adipose tissues mainly modulate metabolic indicators, nonetheless, the visceral adipose tissues as well as the intermuscular adipose tissue were mainly associated with the impaired inflammatory and immune response.

**Conclusions:**

The present study provided the evidence of gene expression profiling that the subcutaneous adipose tissues are mainly affected the metabolism process, whereas the visceral and intermuscular adipose tissues should been term as the metabolic risk factors of obesity.

## Background

Obesity is becoming a major global health hazard mainly because of the increased ingestion of high energy food and the decreased amounts of physical activity in populations. A gain in the adipose mass in the body is generally accompanied by increased risk of metabolism-related diseases. However, it is not only the amount of body fat mass, but also the distribution of adipose tissue that is now recognised as an important predictor of metabolic abnormalities.

Based on their anatomical location, adipose tissue can be divided into two major types: visceral adipose tissue (VAT) and subcutaneous adipose tissue (SAT). The VAT is a higher risk factor for obesity than SAT [1]. In particular, basal free fatty acid flux, lipolysis rate, and secretory protein expression are markedly higher in visceral adipocytes compared with subcutaneous adipocytes [2]. Our previous reports have provided epigenetic evidence that VAT is mainly associated with impaired inflammatory and immune responses and is a metabolic risk factor of obesity, while SAT is mainly associated with metabolic processes [3,4]. In addition, ethnic group and gender are important factors that affect adipose deposition [5,6].

Pig (*Sus scrofa*) has been recognised as an attractive biomedical model for human because its physiology and anatomy are remarkably similar to humans and pigs are easy to breed and handle [7]. Here, we present a comprehensive genome-wide comparison of gene expression profiling among six adipose tissues from different body sites in both sexes of three well-defined pig breeds displaying distinct adipose phenotypes. Using microarray technology, we identified various differentially expressed genes that may be related to the different adipose deposition phenotypes among the breeds, and between the sexes and anatomic locations. We also used a clustering method to identify sets of co-expressed genes that may be functionally linked to different obesity-related phenotypes. These results can be used to understand the different accumulation mechanisms of the distinct adipose tissue types.

## Results and discussion

### Obesity-related phenotypes

Our previous report based on same individuals have demonstrated that the adipose volume were significant difference among adipose tissues and breeds, and between sexes [3]. In addition, the 24 kinds of reprehensive metabolism indicators in serum also revealed the same ranking from the leaner Landrace, the wild Tibetan and the fatty Rongchang pigs [3].

### Identification of differentially expressed (DE) genes and relatedness of adipose samples

**Figure 1:**
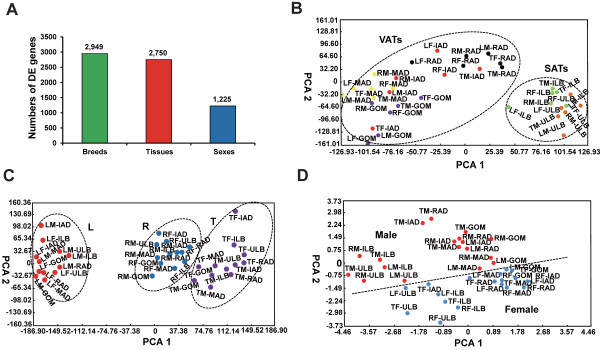
**Identification and the PCA of the DE genes.** (**A**) DE genes across breeds, sexes, and tissues; Two-way PCA of (**B**) six adipose tissues; (**C**) three pig breeds; (**D**) sexes. L, R and T refer to the Landrace, Rongchang and Tibetan, respectively.

The number of DE genes (*P* < 0.05, three-way repeated-measures analysis of variance [ANOVA]) was lowest between male and female (1,225), higher among different adipose tissues (2,750), and highest among the different breeds (2,949), indicating significant biological differences between the groups in the latter two categories (Figure 1A). To further characterise variability in the gene expression profiling, we performed a principal components analysis (PCA) based on the DE genes in each of the compared groups. The six adipose tissues clustered into two groups, VATs and SATs, which reflected their distinct functional and metabolic features (Figure 1B). Interestingly, the intermuscular adipose (IAD) tissue that is deposited between muscle bundles, clustered with the VATs in terms of gene expression profiling, suggesting that IAD may be a potential new risk factor for obesity-related diseases, in agreement with our findings from a previous study on methylation [4].

Although the three pig breeds clustered into three groups, the Rongchang and Tibetan breeds were closer to each other than the Landrace breed (Figure 1C). These results not only reflect the marked phenotypic differences in adipose deposition among the three pig breeds which have undergone different/opposite breeding directions, but also highlight the deep phylogenetic split between the European (Landrace) and Asian pigs (Rongchang and Tibetan) [3].

In addition, the male and female were also been roughly distinguish (Figure 1D), which consisted with the lowest variation of gene expression profiling between sexes, which only have less than half of the amount of DE genes than among tissue types and breeds.

### DE genes between the VATs and SATs

The PCA classified six adipose tissues into two groups: the VAT group which included greater omentum adipose (GOM), mesenteric adipose (MAD), retroperitoneal adipose (RAD) and IAD; and the SAT group which included upper layer of backfat (ULB) and inner layer of backfat (ILB).

**Figure 2:**
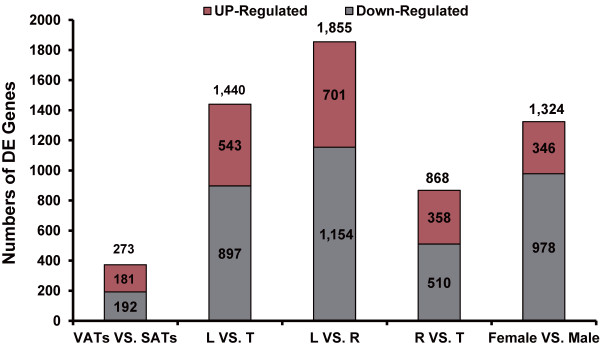
**Identification of up- and down-regulated genes.** L, R and T refer to the Landrace, Rongchang and Tibetan pig, respectively. SAT: subcutaneous adipose tissue; VAT: visceral adipose tissue.

**Figure 3:**
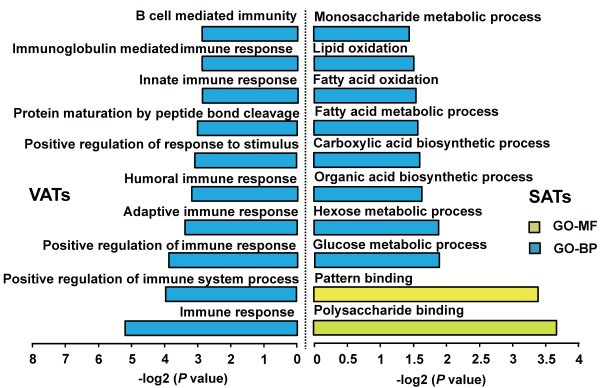
**Top ten GO (Gene Ontology) terms enriched for the up- and down-regulated genes between the SATs and VATs.** The *p* values indicated the significance of the comparison, was calculated by Benjamini-corrected modified Fisher’s exact test.

**Figure 4:**
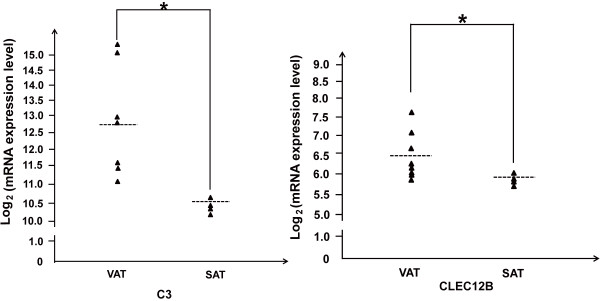
**Differences in mRNA expression levels of the *C3 *and *CLEC12B *gene between the VATs and SATs.**

We performed a pairwise comparison analysis and identified 192 and 181 DE genes that were specifically up-regulated in the SAT and VAT groups, respectively (Figure 2 and Table 1). The DE genes which were specifically up-regulated in the VAT group were mainly enriched in the GO categories of the immune processes, including positive regulation of defence response (8 genes, *P* = 5.63 × 10^-4^), positive regulation of immune system process (10 genes, *P* = 1.11 × 10^-4^), adaptive immune response (6 genes, *P* = 3.54 × 10^-4^), innate immune response (7 genes, *P* = 7.86 × 10^-4^) and B cell mediated immunity (5 genes, *P* = 9.91 × 10^-4^) (Figure 3). Various genes known to be involved in the immune response were highly expressed in VATs compared with in SATs (*P* < 0.05). For example, *C3* (complement 3), which was reported to play a central role in the activation of the complement system [8,9] was up-regulated in the VATs. *CLEC12B* (C-type lectin domain family 12B) limits the activity of monocyte-derived immune cells after cell differentiation and possibly during inflammatory diseases [10] (Figure 4). The action of these genes which were highly expressed in VATs may contribute to the obesity-induced chronic inflammation in adipose tissue that precedes the development of insulin resistance and type II diabetes.

**Table 1:** Identification of DE genes which specifically up-regulated in SAT and VAT groups

**Libraries**	**Up-regulated genes**	**Down-regulated genes**
ULB VS. GOM	298	378
ULB VS. MAD	304	291
ULB VS. RAD	164	142
ULB VS. IAD	154	163
ILB VS. GOM	286	302
ILB VS. MAD	260	222
ILB VS. RAD	73	60
ILB VS. IAD	84	119
Specific expression genes	192 (>4) *	181 (>4) *

*: if a DE gene were identified from at least four comparisons, which been termed as the tissue- specific DE gene.

The DE genes that were specifically up-regulated in the SAT group were mainly enriched in the GO categories of metabolic processes, including glucose metabolic process (6 genes, *P* = 1.16 × 10^-2^), hexose metabolic process (6 genes, *P* = 2.81 × 10^-2^), fatty acid metabolic process (6 genes, *P* = 3.15 × 10^-2^) and monosaccharide metabolic process (6 genes, *P* = 4.77 × 10^-2^) (Figure 3). This result is consistent with previous reports that, compared with the high-risk of visceral obesity caused by VATs, SATs are mainly associated with metabolic processes [4,11].

### DE genes among the three pig breeds

The number of DE genes (*P* < 0.05, three-way repeated-measures ANOVA) was highest between the fatty Rongchang and leaner Landrace pigs (1,855), lower between the wild Tibetan and Landrace pigs (1,440), and lowest between the Tibetan and Rongchang pigs (868) (Figure 2).

**Figure 5:**
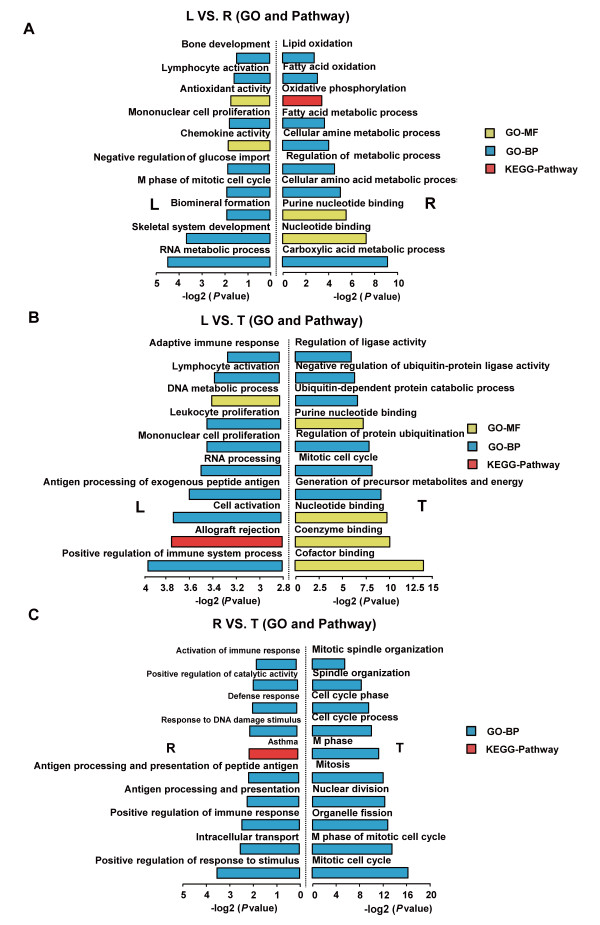
**Top ten GO (Gene Ontology) terms enriched for the up- and down-regulated genes among the three pig breeds.** (**A**) The Landrace versus Rongchang pig; (**B**) The Landrace versus Tibetan pig; (**C**) The Rongchang versus Tibetan pigs. The *P* values indicated the significance of the comparison, was calculated by Benjamini-corrected modified Fisher’s exact test.

The highly expressed genes in the Landrace pigs were mainly involved in the GO and KEGG categories of skeletal system development processes and immune processes, including positive regulation of skeletal system development (7 genes, *P* = 2.22 × 10^-4^), bone development (8 genes, *P* = 4.16 × 10^-2^), positive regulation of immune system process (20 genes, *P* = 1.19 × 10^-3^) and adaptive immune response (8 genes, *P* = 2.39 × 10^-2^) (Figure 5). The highly expressed genes in the Rongchang pigs were mainly involved in the processes of lipid metabolism and immune processes, such as lipid oxidation (8 genes, *P* = 1.40 × 10^-3^), fatty acid oxidation (8 genes, *P* = 2.53 × 10^-3^), fatty acid metabolic process (21 genes, *P* = 4.71 × 10^-4^), activation of immune response (6 genes, *P* = 1.30 × 10^-2^) and positive regulation of immune response (8 genes, *P* = 5.93 × 10^-3^). This result reflects the phenotypic divergence of adipose deposition between these two pig breeds that have undergone artificial selection for different breeding targets. The Landrace breed is under strong artificial selection for more muscle production, while the Rongchang breed is selected for more adipose deposition.

### DE genes between the sexes

**Figure 6:**
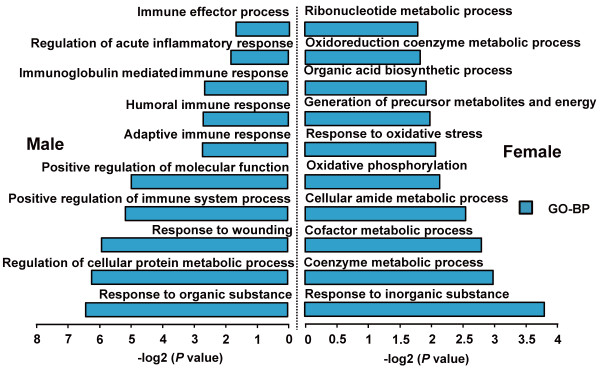
**Top ten GO (Gene Ontology) terms enriched for the up- and down-regulated genes between the male and female pig breeds.** The *p* values indicated the significance of the comparison, was calculated by Benjamini-corrected modified Fisher’s exact test.

Almost three-quarters of the DE genes between the sexes (879 of 1,225, 71.76%) were up-regulated in the males (Figure 2). These highly expressed genes were enriched mainly in the GO categories of the immune processes (Figure 6), including positive regulation of immune system process (28 genes, *P* = 4.49 × 10^-6^), adaptive immune response (11 genes, *P* = 1.71 × 10^-3^), humoral immune response (7 genes, *P* = 1.86 × 10^-3^), immunoglobulin mediated immune response (9 genes, *P* = 2.09 × 10^-3^), regulation of acute inflammatory response (5 genes, *P* = 1.15 × 10^-2^) and immune effector process (13 genes, *P* = 1.33 × 10^-2^). The DE genes (346 of 1,225, 28.24%), which were up-regulated in the females (Figure 2), were mainly involved in metabolic processes (Figure 6), including the coenzyme metabolic process (10 genes, *P* = 1.08 × 10^-3^), cofactor metabolic process (11 genes, *P* = 1.64 × 10^-3^), oxidoreduction coenzyme metabolic process (5 genes, *P* = 1.54 × 10^-2^) and organic acid biosynthetic process (8 genes, *P* = 1.10 × 10^-2^). This result reflects a sexual dimorphism in body fat distribution [12]. Females deposit relatively more fat in subcutaneous/inguinal depots, which has beneficial effects on the control of body weight and metabolism [13]; on the other hand, males tend to deposit more fat in intra-abdominal/gonadal depots, which has been found to be related to risk for a number of diseases [14].

**Figure 7:**
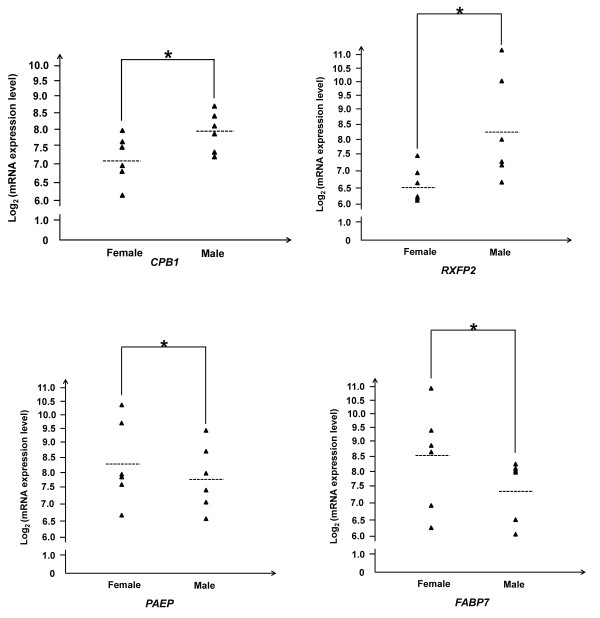
**Differences in mRNA expression levels of the *CPB1, RXFP2, PAEP *and *FABP7 *gene between the between the females and males.**

Interestingly, we identified some genes that potentially contribute to the sexual differences in obesity development. Compared with the females, the males exhibited higher expression levels of *RXFP2* (*P* =1.19 × 10^-13^) and *CPB1* (*P* = 5.69 × 10^-6^) (Figure 7), which have been related to gubernaculum testis development and spermatogenesis [15,16]. However, compared with the males, the females exhibited higher expression levels of *PAEP* (*P* = 3.71 × 10^-5^) and *FABP7* (*P* = 8.42 × 10^-9^) (Figure 7), which have been associated mainly with the secretory endometrial glands and breast cancer [17,18].

### Validation of gene expression changes by Q-PCR

**Figure 8:**
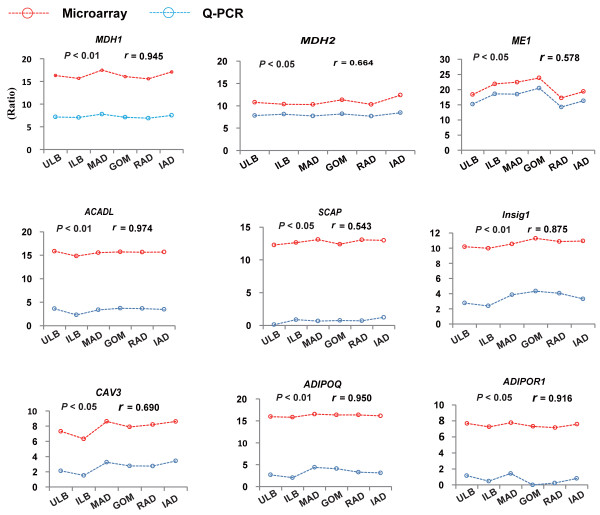
**Validation of gene expression by Q-PCR.** The Pearson correlation coefficient (*r*) and the corresponding significance value (*P*) were shown above the columns.

The expression changes of nine genes that are well-known to be related to adipose metabolism (*ACADL*, *ADIPOQ*, *ADIPOR 1*, *CAV 3*, *MDH 1*, *MDH 2*, *ME 1*, *Insig 1* and *SCAP*) showed significant positive correlations between the Q-PCR results and their expressions determined by microarray (*P* < 0.05, Figure 8), which highlighted the high confidence of the results from the microarray approach.

### Co-expressed gene sets linked to the obesity-related phenotypes

Generally, quantitative characters (such as adipose deposition) in mammals are determined by a set of genes that cooperate in certain biological processes [11]. To further explore the relationships between the genes and obesity-related traits, we applied a clustering method to identify sets of functionally related genes linked to the phenotypic traits as reported previously [19].

**Figure 9:**
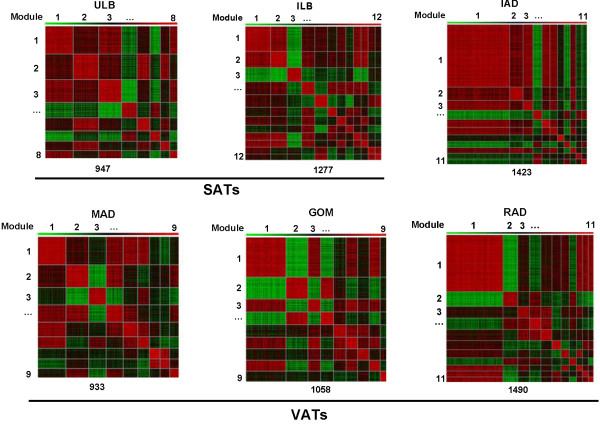
**Heat maps of co-expressed gene modules in six adipose tissues.** Two SATs (ILB: inner layer of backfat, ULB: upper layer of backfat); three VATs (GOM: greater omentum, MAD: mesenteric adipose, RAD: retroperitoneal adipose); and intermuscular adipose (IAD). Pair-wise correlations between genes residing in all the gene sets were plotted. Bright red indicates a strong positive co-expression, whereas bright green indicates strong negative co-expression.

We identified eight gene modules containing 947 genes for ULB and twelve gene modules containing 1,277 genes for ILB. In three typical VATs, we identified nine, nine and eleven gene modules containing 933, 1,058 and 1,409 genes for the MAD, GOM and RAD tissues, respectively. For the IAD, we identified eleven modules containing 1,423 genes (Figure 9).

**Figure 10:**
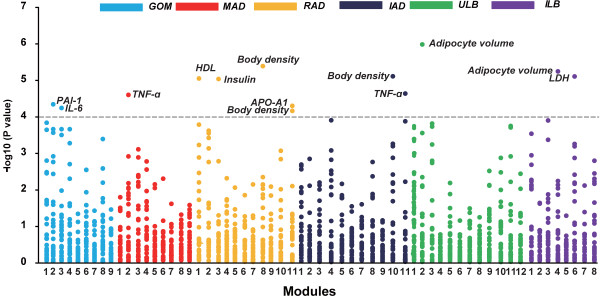
**Correlations between co-expressed gene modules in six adipose tissues and obesity phenotypic traits.** -Log10 *P* values for Spearman rank correlation coefficients between the expressed values of the gene modules and the different obesity phenotypic traits are shown. The gray line represents the Bonferroni corrected *P* value greater than 0.05.

Next, we explored the correlations between the modules of co-expressed genes and the various obesity phenotypes. Three gene modules in the SATs (i.e. ULB and ILB) were significantly correlated with adipocyte volume and the concentration of plasma lactate dehydrogenase in serum (false discovery rate adjusted permutation, *P* < 0.05) (Figure 10 and Table 2). The co-expressed genes within these modules were mainly involved in metabolic processes, such as fatty acid metabolic process (23 genes, *P* = 4.38 × 10^-5^), fatty acid beta-oxidation (3 genes, *P* = 2.65 × 10^-3^), coenzyme metabolic process (6 genes, *P* = 4.77 × 10^*-5*^*)* and carbohydrate metabolic process (12 genes, *P* = 2.94 × 10^-3^) (Table 3), in agreement with an earlier report that SATs contribute mainly to metabolic processes [20,21].

**Table 2:** Modules correlated with the obesity-related traits in SATs and VATs

**Tissues**		**Module ID**	**Correlated trait**	**No. of genes in module**	**Correlation coefficient**	***P*-value**
**SAT**	**ULB**	4	Adipocyte volume	110	−0.85	8.12 × 10^-6^
		6	LDH	72	−0.84	1.23 × 10^-5^
	**ILB**	2	Adipocyte volume	157	0.87	2.05 × 10^-6^
**VAT**	**GOM**	2	PAI-1	162	−0.81	4.97 × 10^-5^
		3	IL-6	92	−0.80	6.28 × 10^-5^
	**MAD**	2	TNF-a	152	0.82	2.57 × 10^-5^
	**RAD**	8	Body density	71	0.87	2.97 × 10^-6^
		11	Body density	51	−0.81	5.44 × 10^-5^
		1	HDL	590	0.85	7.42 × 10^-6^
		11	Apo-A1	51	−0.81	3.91 × 10^-5^
		3	Insulin	118	0.77	6.89 × 10^-6^
	**IAD**	10	Body density	55	0.86	5.90 × 10^-6^
		11	TNF-a	52	−0.83	2.15 × 10^-5^

**Table 3:** Top ten GO (Gene Ontology) and pathway categories enriched for co-expressed gene modules which correlated with obesity phenotypic traits

**Correlated trait**	**Tissue (Gene sets order No.)**	**Functional category**	**Term**	***P*values**	**Involved gene No.**
**Adipocyte volume**	ULB (4)	GO-BP	metabolic process	1.19 × 10^-7^	119
	ILB (2)	GO-BP	coenzyme metabolic process	8.84 × 10^-6^	8
		GO-BP	lipid metabolic process	4.38 × 10^-5^	23
		GO-BP	fatty acid metabolic process	8.95 × 10^-5^	10
		GO-BP	primary metabolic process	1.07 × 10^-4^	104
		GO-BP	translation	5.37 × 10^-4^	12
		GO-BP	protein folding	1.50 × 10^-3^	8
		GO-BP	fatty acid beta-oxidation	2.65 × 10^-3^	3
		GO-BP	pentose-phosphate shunt	3.51 × 10^-3^	2
		GO-BP	generation of precursor metabolites and energy	4.15 × 10^-3^	9
**Body density**	RAD (8,11)	GO-BP	translation	1.44 × 10^-3^	8
	IAD (10)	GO-BP	protein targeting	1.90 × 10^-3^	5
		GO-BP	lysosomal transport	5.44 × 10^-3^	2
		GO-MF	structural constituent of ribosome	6.10 × 10^-3^	5
		GO-MF	structural molecule activity	6.62 × 10^-3^	15
		GO-BP	nitrogen utilization	1.20 × 10^-2^	1
		KEGG	Serine glycine biosynthesis	2.97 × 10^-2^	1
		GO-BP	regulation of amino acid metabolic process	3.55 × 10^-2^	1
		GO-BP	pyrimidine base metabolic process	4.40 × 10^-2^	2
		GO-BP	pentose-phosphate shunt	4.71 × 10^-2^	1
**Apo-A1 (Metabolic indicators)**	RAD (11)	GO-BP	nitrogen utilization	3.51 × 10^-3^	1
		GO-BP	regulation of amino acid metabolic process	1.05 × 10^-2^	1
		GO-BP	lysosomal transport	3.12 × 10^-2^	1
		GO-BP	nitrogen compound metabolic process	3.12 × 10^-2^	1
		GO-BP	neuromuscular synaptic transmission	4.64 × 10^-2^	1
		GO-MF	hormone activity	1.55 × 10^-2^	2
		KEGG	Vasopressin synthesis	2.09 × 10^-2^	1
		KEGG	De novo pyrmidineribonucleotidesbiosythesis	3.12 × 10^-2^	1
		KEGG	Cortocotropin releasing factor receptor signaling pathway	5.14 × 10^-2^	1
		KEGG	De novo purine biosynthesis	5.64 × 10^-2^	1
**HDL (Metabolic indicators)**	RAD (1)	GO-BP	generation of precursor metabolites and energy	1.20 × 10^-15^	37
		GO-BP	metabolic process	1.49 × 10^-14^	273
		GO-BP	respiratory electron transport chain	1.35 × 10^-11^	30
		GO-BP	coenzyme metabolic process	3.02 × 10^-9^	16
		GO-BP	primary metabolic process	9.31 × 10^-9^	241
		GO-BP	signal transduction	6.95 × 10^-7^	48
		GO-BP	cell communication	8.92 × 10^-7^	52
		GO-BP	cell surface receptor linked signal transduction	1.49 × 10^-6^	15
		GO-BP	system development	3.58 × 10^-6^	9
		GO-BP	tricarboxylic acid cycle	5.49 × 10^-6^	7
**LDH (Metabolic indicators)**	ULB (3)	GO-BP	fatty acid metabolic process	1.37 × 10^-5^	9
		GO-BP	metabolic process	1.86 × 10^-5^	75
		GO-BP	fatty acid beta-oxidation	3.05 × 10^-5^	4
		GO-BP	coenzyme metabolic process	4.77 × 10^-5^	6
		GO-BP	cellular amino acid biosynthetic process	9.01 × 10^-5^	5
		GO-BP	cellular amino acid metabolic process	1.05 × 10^-4^	9
		GO-BP	protein folding	4.66 × 10^-4^	7
		GO-BP	pentose-phosphate shunt	1.45 × 10^-3^	2
		GO-BP	primary metabolic process	1.99 × 10^-3^	66
		GO-BP	carbohydrate metabolic process	2.94 × 10^-3^	12
**Insulin (Metabolic indicators**	RAD (3)	KEGG	VEGF signaling pathway	3.72 × 10^-4^	4
		GO-BP	transcription, RNA-dependent	4.06 × 10^-4^	2
		GO-BP	cell communication	6.62 × 10^-4^	31
		GO-BP	mesoderm development	1.04 × 10^-3^	11
		GO-BP	cellular process	1.10 × 10^-3^	40
		GO-BP	protein amino acid ADP-ribosylation	1.11 × 10^-3^	2
		GO-BP	signal transduction	1.37 × 10^-3^	29
		GO-BP	esicle-mediated transport	1.69 × 10^-3^	11
		GO-BP	system development	1.93 × 10^-3^	14
		GO-BP	cell surface receptor linked signal transduction	4.81 × 10^-3^	16
**IL-6 (Inflammatory and immune adipokines)**	GOM (3)	GO-BP	immune system process	6.66 × 10^-4^	21
	IAD (11)	GO-BP	response to stimulus	7.72 × 10^-4^	16
		GO-BP	response to interferon-gamma	2.08 × 10^-3^	4
		GO-BP	cellular defense response	4.67 × 10^-3^	6
		GO-BP	immune response	6.84 × 10^-3^	8
		GO-BP	DNA recombination	1.06 × 10^-2^	3
		GO-BP	nitrogen utilization	1.16 × 10^-2^	1
		GO-BP	gluconeogenesis	1.35 × 10^-2^	2
		GO-BP	antigen processing and presentation of peptide or polysaccharide antigen via MHC class II	1.81 × 10^-2^	2
		KEGG	T cell activation	1.84 × 10^-2^	4
**TNF-*α*(Inflammatory and immune adipokines)**	MAD (2)	GO-BP	antigen processing and presentation of peptide or polysaccharide antigen via MHC class II	4.26 × 10^-5^	4
		GO-BP	antigen processing and presentation	6.56 × 10^-4^	4
		KEGG	T cell activation	1.37 × 10^-3^	4
		GO-BP	cellular defense response	3.14 × 10^-3^	6
		GO-BP	immune system process	8.14 × 10^-3^	17
		GO-BP	tRNAaminoacylation for protein translation	2.18 × 10^-2^	2
		KEGG	5-Hydroxytryptamine biosynthesis	3.17 × 10^-2^	1
		KEGG	Mannose metabolism	3.69 × 10^-2^	1
		GO-BF	establishment or maintenance of chromatin architecture	3.85 × 10^-2^	4
**PAI-1 (Inflammatory and immune adipokines)**	GOM(2)	GO-BP	immune system process	6.00 × 10^-15^	41
		GO-BP	response to stimulus	6.31 × 10^-15^	30
		GO-BP	cellular defense response	1.25 × 10^-10^	15
		GO-BP	immune response	1.35 × 10^-10^	19
		GO-BP	B cell mediated immunity	1.05 × 10^-7^	9
		GO-BP	natural killer cell activation	4.17 × 10^-6^	7
		GO-BP	antigen processing and presentation	6.62 × 10^-6^	6
		KEGG	T cell activation	2.04 × 10^-5^	6
		GO-BP	cell surface receptor linked signal transduction	1.52 × 10^-4^	23
		KEGG	Inflammation mediated by chemokine and cytokine signaling pathway	3.11 × 10^-4^	8

In all tests, the unified set of co-expressed genes for different adipose depots correlated to an obesity phenotypic trait was compared with the whole known genes which were appointed as the background. *P* values indicating significance of the overlap between various genes sets, was calculated using Benjamini-corrected modified Fisher’s exact test. *BP* biological process, *MF* molecular function.

The co-expressed genes in two of the VATs (i.e. GOM and MAD) mainly affected the concentrations of inflammatory and immune adipokines in serum, i.e. plasminogen activator inhibitor-1 (PAI-1), interleukin 6 (IL-6) and tumor necrosis factor-α (TNF-α) (Figure 10 and Table 2). The co-expressed genes within these modules were mainly involved in immune system process (21 genes, *P* = 6.66 × 10^-4^), immune response (8 genes, *P* = 6.84 × 10^-3^), cellular defence response (15 genes, *P* =1.25 × 10^-10^), cellular defence response (6 genes, *P* = 4.67 × 10^-3^) and B cell mediated immunity (9 genes, *P* = 1.05 × 10^-7^). This result suggested that the GOM and MAD tissues contributed mainly to the obesity-induced chronic inflammation in adipose tissue that precedes the development of insulin resistance and type II diabetes [22,23]. The RAD tissue, on the other hand, was found to affect mainly the body density and the concentrations of three metabolic indicators in serum, namely, high density lipoprotein, insulin, and apolipoprotein A-1 (Apo-A1), which are mainly involved in metabolic processes such as coenzyme metabolic process (16 genes, *P* =3.02 × 10^-9^), primary metabolic process (241 genes, *P* = 9.31 × 10^-9^), and tricarboxylic acid cycle (7 genes, *P* = 5.49 × 10^-6^) (Table 3). Studies in human showed that GOM was strongly correlated to insulin, while no such correlations were found between lipid uptake and SAT or RAD [24].

Similar to the findings for the high-risk VATs, the co-expressed genes in IAD were also found to be significantly correlated with immune-related TNF-α (Figure 10 and Table 2), further suggesting that the IAD is an independent risk factor for metabolic diseases [25].

## Conclusions

The present study describes a genome-wide analysis of gene expression profiling among various adipose depots between the sexes of three well-defined pig breeds displaying distinct adipose phenotypes. We present evidence that the SATs mainly affect metabolic processes, while the VATs and the IAD are associated mainly with immune and inflammation responses that are seen as the metabolic risk factors of obesity. This study also supports pig as an ideal model for studying human obesity.

## Materials and methods

### Tissue collection

Three females and three males at 210-days-old for each of the leaner Landrace pigs (a Western breed), the wild Tibetan pigs (a feral, indigenous Chinese pig that has not undergone artificial selection) and the fatty Rongchang pigs (a Chinese breed) were used in this study as previously described [3]. Animals were humanely sacrificed, in compliance with experimental animals established by the Ministry of Agriculture of China. Three VATs (i.e. GOM, MAD and RAD), two SATs (i.e. ULB and ILB) as well as IAD were rapidly separated from each carcass, immediately frozen in liquid nitrogen, and stored at −80°C until RNA extraction. For more information, please refer to Li *et al*. [3].

### Measurements of the obesity-related phenotypes

Measurements of the pig body density, the concentrations of 24 serum-circulating indicators of metabolism and the adipocyte volume are from our previous report based on same individuals. For more information, please refer to Li *et al.*[3].

### RNA isolation and microarray analysis

The total RNA (10 μg) of the 108 tissue samples was extracted with TRIzol (Invitrogen) and further purified using an Rneasy column (Qiagen). Hybridization, washing, and scanning were done according to standard Agilent protocols. The data analysis was performed with MultiExperiment Viewer (MeV) [24]. Array data have been uploaded to NCBI’s Gene Expression Omnibus (GEO) [accession number GSE30343]. For more information, please refer to Li *et al.*[3].

### Identification of differentially expressed genes

We used three-way repeated-measures ANOVA (*n* = 3 per breed per sex per tissue) to identified differentially expressed (DE) mRNA among six adipose tissues between sexes of three pig breeds (*P* < 0.05, corrected with adjusted Bonferroni method, FDR <0.05, 1,000 permutations), which were further analyzed using DAVID to examine whether these DE genes were enriched for specific functional catalogs (i.e. KEGG pathways and Gene Ontology-Biological Processes and -Molecular Functions) (*P* < 0.05, corrected with adjusted Bonferroni method) [26].

### Identification of co-expressed gene modules

To identify sets of functionally related genes in six adipose tissues, linked to the phenotypic traits, we applied a clustering method as described previously [4,19] with some modifications. Spearman rank correlation coefficients were determined between all possible gene-pairs across 18 samples for each adipose tissue. The strongest correlated gene-pair was selected, and grouped together in a set that was assigned the average expression value of the two genes that constitute this set. After addition of this newly created set to the dataset, the two individual genes were removed from the data and the strongest correlation in the dataset was again selected. This resulted in either the expansion of a set already created or in the creation of a new set. We kept repeating this as an iterative process until the most significantly correlated pair was coefficient of correlation *r* < 0.80. Only the sets containing 50 or more genes are kept for further analysis. The gene sets - reflecting the average expression value of the genes constituting that set - were correlated with obesity phenotypic traits using a Spearman rank correlation coefficient.

### Quantitative PCR

Quantitative PCR (Q-PCR) was used to confirm the expression pattern observed in the microarray results. Q-PCR was performed on a CF96 Real-Time PCR Detection System (Bio-Rad) using SYBR® Green Real-time PCR Master Mix (TaKaRa, China). The PCR primer sequences are shown in Table 4. Porcine *ACTB*, *TBP* and *TOP2B* were simultaneously used as endogenous control genes. The 2^-ΔΔCt^ method was used to determine the relative mRNA abundance for the surveyed samples.

**Table 4:** Primers used for the Q-PCR assay

**Gene symbol**	**Primer sequence (5′ → 3′)**	**Amplicon length (bp)**	**GenBank No.**
ACTB*	F: TCTGGCACCACACCTTCT	114	DQ178122
	R: TGATCTGGGTCATCTTCTCAC		
TBP*	F: GATGGACGTTCGGTTTAGG	124	DQ178129
	R: AGCAGCACAGTACGAGCAA		
TOP2B*	F: AACTGGATGATGCTAATGATGCT	137	AF222921
	R: TGGAAAAACTCCGTATCTGTCTC		
***ACADL***	F: GTAAGAACAAATGCCAAGA	103	NM_213897
	R: CAGCCACTACAATCACAAC		
***ADIPOQ***	F: GGGTCACTGTCCCTAAC	209	NM_214370
	R: GTCCTGGTACTGGTCGT		
***ADIPOR1***	F: TGGCTGAAGGACAACGAC	228	NM_001007193
	R: CAAGAAGAACATCCCAAACAC		
***CAV3***	F: GCATCAGCCATATCTACTCACT	107	NM_001037149
	R: CACTTCTTTCCGCAGCAT		
***MDH1***	F: TAAGGTTATCGTGGTGGG	124	U44846
	R: TGCTTTAGCTCGGTTGTG		
***MDH2***	F: CGAGGTGGTCAAGGCTAAG	172	M16427
	R: CAATGGCGTGGAGAAATAC		
***ME 1***	F: GTTGCCCTTGGTGTTGT	212	X93016
	R: GGATAAATGGTGGCTGTC		
***Insig1***	F: TCAACCACGCCAGTGCTAA	251	AY336601.2
	R: CACGCCTCCCGAGAAGAAA		
***SCAP***	F: CGCCTGAAACAGAAATCG	213	XM_001924223
	R: TCTCCTGAGCCTCCAACA		

*: *ACTB* (β actin), *TBcP* (TATA box binding protein) and *TOP2B* (topoisomerase II β) are the endogenous control genes.

## Abbreviations

SAT: Subcutaneous adipose tissue; VAT: Visceral adipose tissue; ULB: Upper layer of backfat; ILB: Inner layer of backfat; GOM: Greater omentum adipose; MAD: Mesenteric adipose; RAD: Retroperitoneal adipose; IAD: Intermuscular adipose.

## Competing interests

The authors have declared that no competing interests exist.

## Authors’ contributions

MZL and XWL conceived and designed the experiment. CWZ and MZL performed the data analysis and drafted the manuscript. JDM and AAJ collected the samples and records. GQT, MMM, LZ and LB carried out the physiological, biochemical and histological studies, statistical analysis and prepared nucleic acids. CWZ and JZ performed gene expression microarray and data analysis. All authors read and approved the final manuscript.
